# MicroRNA Signature of Traumatic Brain Injury: From the Biomarker Discovery to the Point-of-Care

**DOI:** 10.3389/fneur.2018.00429

**Published:** 2018-06-14

**Authors:** Valentina Di Pietro, Kamal M. Yakoub, Ugo Scarpa, Cinzia Di Pietro, Antonio Belli

**Affiliations:** ^1^Neurotrauma and Ophthalmology Research Group, Institute of Inflammation and Ageing, University of Birmingham, Birmingham, United Kingdom; ^2^Surgical Reconstruction and Microbiology Research Centre, National Institute for Health Research, Queen Elizabeth Hospital, Birmingham, United Kingdom; ^3^Beckman Institute for Advanced Science and Technology, University of Illinois at Urbana-Champaign, Illinois, IL, United States; ^4^BioMolecular, Genome and Complex Systems BioMedicine Unit, Section of Biology and Genetics G Sichel, Department of Biomedical Sciences and Biotechnology, University of Catania, Catania, Italy

**Keywords:** traumatic brain injury, biomarkers, microRNA, diagnosis, prognosis, therapy, high-throughput technology, point-of-care

## Abstract

Traumatic brain injury (TBI) is a serious problem that causes high morbidity and mortality around the world. Currently, no reliable biomarkers are used to assess the severity and predict the recovery. Many protein biomarkers were extensively studied for diagnosis and prognosis of different TBI severities such as S-100β, glial fibrillary acidic protein (GFAP), neuron-specific enolase (NSE), neurofilament light chain (NFL), cleaved tau protein (C-tau), and ubiquitin C-terminal hydrolase-L1 (UCH-L1). However, none of these candidates is currently used in the clinical practice, due to relatively low sensitivity, for the diagnosis of mild TBI (mTBI) or mild to moderate TBI (MMTBI) patients who are clinically well and do not have a detectable intracranial pathology on the scans. MicroRNAs (miRNAs or miRs) are a class of small endogenous molecular regulators, which showed to be altered in different pathologies, including TBI and for this reason, their potential role in diagnosis, prognosis and therapeutic applications, is explored. Promising miRNAs such as miR-21, miR-16 or let-7i were identified as suitable candidate biomarkers for TBI and can differentiate mild from severe TBI. Also, they might represent new potential therapeutic targets. Identification of miRNA signature in tissue or biofluids, for several pathological conditions, is now possible thanks to the introduction of new high-throughput technologies such as microarray platform, Nanostring technologies or Next Generation Sequencing. This review has the aim to describe the role of microRNA in TBI and to explore the most commonly used techniques to identify microRNA profile. Understanding the strengths and limitations of the different methods can aid in the practical use of miRNA profiling for diverse clinical applications, including the development of a point-of-care device.

## MicroRNA signature in traumatic brain injury

### Traumatic brain injury

Head injuries are a significant cause of disability and mortality worldwide and one of the most common reasons of emergency department visits especially among young males (1), creating a severe physical, psychological and socioeconomic burden on the patients, their families and the community (1, 2).

In particular, traumatic brain injury (TBI) is a complex pathological alteration in the neural homeostasis which is triggered by an external mechanical force resulting in a broad spectrum of temporary or permanent injuries and outcomes (3). Annual TBI incidents are estimated to be more than 10 million patients worldwide (4–6). The most frequent causes of TBI are falls, road traffic accidents, sport and recreation activities, military injuries and assault or abuse. TBI pathology can be classified as primary and secondary brain damage (7). The primary injury occurs immediately after receiving the mechanical impact which disrupts the integrity of neuronal, glial, endothelial cells and dysregulates the cerebral blood flow (CBF), whereas the secondary brain injury is due to a range of biochemical and cellular changes that causes neuronal apoptosis and death, blood-brain barrier (BBB) disruption, etc. (8–15). Controlling the development of secondary injury is the only strategy that can be beneficial to improve the outcome of the primary injury that cannot be managed medically. The heterogeneity of the disease makes an accurate assessment of the severity of trauma and the prediction of patient outcome, challenging. Clinically, head injuries are diagnosed as mild, moderate, or severe according to the Glasgow Coma Scale (GCS) score, which uses a motor, eye and verbal responses to assess the conscious level of the patient. However, this score might underestimate mild TBI (mTBI) cases (16). Computed tomography (CT) or magnetic resonance imaging (MRI) scans are also used to assess TBI according the current guidelines (17). Although these techniques show limited diagnostic ability for the detection of mild brain tissue insult with concerns for radiation risks from CT scans and the escalating costs of diagnostic imaging techniques (18, 19) in the future, imaging has the potential to complement molecular diagnostics (20).

For this reason, mTBI detection remains one of the most difficult clinical diagnoses, it accounts for 75–90% of the TBI cases in the United States (21) and 10–20% of the patients remain symptomatic and complain of post-concussive syndrome (PCS) symptoms (22). In addition, people such as military, sportive and children are at risk of repeated concussions and may develop depression (23) and neurodegenerative conditions in later life, e.g., Parkinson's disease, motor neuron disease, and chronic traumatic encephalopathy (CTE) (24, 25).

### Biomarkers of traumatic brain injury

Currently, no TBI biomarkers were identified that could reliably be used in the clinical practice for diagnosis and prognosis.

Recently, the U.S. Food and Drug Administration reviewed and authorized for marketing the Banyan Brain Trauma Indicators which are ubiquitin C-terminal hydrolase-L1 (UCH-L1) and glial fibrillary acidic protein (GFAP), to evaluate mTBI in adults. These two proteins are released from the injured tissue into the blood and can be quantified within 12 h of the brain injury and can help to predict the patients with detectable intracranial lesions on the CT scan with 97.5% of accuracy. However, a biomarker able to accurately diagnose mTBI is still needed.

In the last decades, many molecules were proposed as promising TBI biomarkers, but the complicated anatomy of the brain and the disparate pathology of the TBI make it challenging to apply into the clinical practice (26).

Biofluid biomarkers would be preferable as they present various advantages such as cost-effective and minimally invasive sample collection.

Among the most extensively studied biomarkers in the serum and cerebrospinal fluid (CSF), there are S-100β and GFAP. S-100β is an extracellular protein with a short half-life of <30 min (27). However, because of its size, it does not cross an intact BBB. Besides, S-100β is not a brain-specific protein and can also be released by other organs in case of polytrauma (28–31). In 2013, the S-100β serum level was used to reduce the unnecessary CT scans in the adult mTBI patients among the Scandinavian population. However, it remains challenging to find the appropriate cut-off value of S-100β that correlates with the injury primarily because of the lower sensitivity in polytrauma patients (32, 33).

On the contrary, GFAP is a structural protein exclusively expressed in the astroglial cells and plays a pivotal role in the astrocyte's cytoskeleton as a component of the intermediate filament (IF) network (34). GFAP was found to be slightly elevated in mild TBI and when added to the clinical data, it improved the power of outcome prediction (35). Animal studies also showed GFAP to be a promising biomarker, since its cellular release is correlated to all grades of injury severities (36). The only limitation in the use of GFAP as a biomarker is related to the release into the bloodstream or CSF, which is, indeed, strictly BBB-damage dependent (35, 37).

Neuron-specific enolase (NSE), neurofilament light polypeptide (NFL), cleaved tau protein (C-tau) and UCH-L1 were also considered promising biomarkers. However, the biological significance of these biomarkers cannot be confidently declared, due to the lack of studies with adequate sample size and low sensitivity for mTBI in individuals without detectable structural brain abnormalities. A summary of papers showing the area under the curve (AUC) of representative TBI biomarkers is presented in Table 1.

**Table 1:** Area under the curve (AUC) of representative TBI biomarkers.

**Biomarkers**	**AUC**	**Cohort**	**Condition**	**N**	**Controls**	**Reference**	**Timing**	**Comment**
S100B	0.87	TBI all severity	TBI vs. non-TBI	50	50	(38)	Within 6 h	Non-specific
S100B	0.68	mTBI	Ice hockey vs. pre-season	28	28	(39)	Within 1 h	Poor performance
NSE	0.82	TBI all severity	TBI vs. non-TBI	50	50	(38)	Within 6 h	Non-specific
NSE	0.54	mTBI		28	28	(39)	Within 1 h	Poor performance
NSE	0.64	mTBI	Clinically important injury	25	82	(40)	Day 1	Non-specific
Myelin-basic protein	0.66	TBI all severity	TBI vs. non-TBI	50	50	(38)	Within 6 h	Poor performance
Cleaved Tau	0.74	mTBI	Injury vs. pre-season	28	28	(41)	At 36 h	Late
Total Tau	0.8	mTBI	Ice hockey vs. pre-season	28	28	(39)	Within 1 h	Promising
GFAP	0.84	mild-moderate TBI	Positive CT	209	188	(42)	At 4 h	Limited sensitivity
UCH-L1	0.87	mTBI	GCS 15 vs. controls	86	199	(43)	Within 1 h	Promising
UCH-L1	0.73	TBI	positive CT	N/A	199	(43)	Within 1 h	Promising
Amyloid-β	N/A	sTBI	TBI vs. controls	12	20	(44)	Day 1	poor sensitivity
All-Spectrin break-down	0.76	mTBI	Injury vs. pre-season	25	N/A	(41)	At 36 h	Late
CTS5	N/A	TBI all severity	sTBI vs. orthopedic injury	30	30	(45)	Within 1 h	Promising

Enolases are glycolytic enzymes composed by three different subunits (α, β, γ). The two most stable isoforms are γγ and αγ, which are referred to as NSE, are particularly abundant in the neuron cytoplasm, however NSE proteins can also be found in erythrocytes and platelets making the process of haemolysis a significant extracranial source when measured in trauma (46, 47). In the context of mild TBI, NSE can predict the early prognosis of patients when measured in combination with S-100β (48). However, its slow elimination from plasma, leads to difficulties in distinguishing between primary and secondary insults to the brain (49, 50).

One of the most recently identified biomarkers is NFL. It was suggested as a potential, sensitive and specific marker in detecting axonal injury in mTBI (51). One of the advantages of its clinical use is the relatively long half-life which was estimated to be ~3 weeks (52). NFL also plays a vital role in the neuro-axonal cytoskeleton (53). Therefore, increased NFL levels were found in the CSF and serum of individuals with a wide range of neurodegenerative and neuroinflammatory diseases (54, 55). Another proposed serum marker is C-tau, which is a microtubule-associated protein (MAP) primarily found in the neuronal axons and dendrites (49, 56). After an axonal injury, tau protein can be detected in the extracellular space and diffuses into the CSF after N- and C- terminals cleavage (56). In 2006, a study demonstrated that higher levels of post-traumatic CSF C-tau were associated with a poorer clinical outcome following severe TBI (sTBI) (57). However, there was no significant correlation between the levels of C-tau and the outcome following mTBI (58).

UCH-L1 was identified as highly specific to the human brain (59) and the increased levels were correlated with the TBI severity and a worse outcome. Its diagnostic value was found to be beyond the first 24 h of injury (60–63). It could also distinguish between the patients with TBI and the uninjured patients with altered GCS secondary to drugs and alcohol intoxication (50).

Recently, a new protein Cystatin D (CTS5), which inhibits lysosomal and secreted cysteine proteases, was also identified as a potential biomarker to assess the severity of TBI and its expression at very early time points, makes CTS5, an ideal biomarker for a point-of-care (PoC) device (45). To the best of our knowledge, none of the previous protein biomarkers has been successfully used in the clinical setting for diagnosis and prognosis of TBI patients.

### MicroRNAs as emerging biomarkers in TBI

MicroRNAs (miRNAs or miRs) are a class of molecular regulators discovered for the first time in *Caenorhabditis elegans* in 1993 (64). Then, dozens of miRNAs were identified in worms, flies and human suggesting that miRNAs represent a previously unknown group of molecules (65).

miRs are short (~22 nucleotides) non-coding, single-stranded RNAs that play key roles in the regulation of several biological processes such as cell proliferation and differentiation, survival, and motility via negative feedback mechanism at the post-transcriptional level by binding to the 3′-untranslated region (UTR) of the target miRs and leading to either suppression of the translation process, mRNAs degradation or both. A single miRNA can regulate multiple mRNAs and vice-versa because they do not always require a perfect complementarity for target recognition. Therefore, they can briefly interchange between the cellular programs (66).

The synthesis of miRs begins in the nucleus with transcription by the RNA polymerase II or III producing long primary miRNA transcripts (pri-miRNA) that contain functional secondary structures, termed stem-loops and carrying mature miRNA sequences. Maturation of the pri-miRNA transcripts includes several steps which are initiated by RNase III endonuclease Drosha and produces the precursor-miR (pre-miR) (67, 68). Following Drosha processing, a complex of proteins, exportin-5 (EXP5) with GTP-binding nuclear protein Ran-GTP, transports pre-miR from the nucleus into the cytoplasm where it is cleaved by Dicer and TAR RNA-binding protein (TRBP) (69, 70). This produces a double-stranded RNA molecule composed of 20–24 nucleotide miR and a complementary miR^*^ of the same length (71). It has been found that not only the mature miR strand is biologically active, but also the miR^*^ strand is functional and not just degraded as was previously hypothesized (72). Then, mature miRNAs bind to mRNA molecules through a process facilitated by the RNA-induced silencing complex (RISC), which consists of RNase Dicer, TRBP, PACT (protein activator of PKR) and the Argonaute proteins (68).

The resulting RISC-miRNA complex binds the complementary regions of the target mRNAs by partial or total base-pairing at the 3′ UTR. This interaction, controlled by nucleotides 2–8 at the 5′ end of the miR and known as “seed sequence” (73), reduces protein production by translation inhibition and mRNA degradation (74). However, miRNAs do not target all mRNAs because there are only binding sites in one-third of the mRNAs (75).

Currently, in the human genome, over 2,000 miRNAs were identified and numerous studies were mainly focused on the miRNA profiling in various tissues and biofluids that can aid the diagnosis of a wide range of diseases, including cancer, cardiovascular, nervous system disorders and many other disorders (76, 77, 147). Since miRs are relatively abundant and stable in the human biofluids, they are considered to be better than protein biomarkers and therefore are now being investigated as the new class of markers for numerous pathologies including but not limited to neurodegenerative diseases. However, a better understanding of the biological mechanisms of miRNAs in these diseases is required to improve their application as biomarkers (78).

With the discovery of miRNAs and its critical role as regulators in various diseases, it is now possible to investigate their role as biomarkers and emerging therapeutic targets. Based on the antisense technology, very potent oligonucleotides targeted against miRNA known as anti-miR were developed (79, 80).

TBI research associated with the changes in miRNA expression is only at the beginning to be understood. Few studies showed the miRNA profile in serum plasma and CSF after different TBI severities and at different time points (81–85).

Redell et al. found a downregulation of miR-16 and miR-92a in severe TBI patients and an upregulation of miR-765 in mild and severe TBI, within the first 24 h and by using a microarray approach (81). Bhomia and collaborators analyzed microRNA profile in serum and CSF of patients grouped in three different categories, mild-moderate TBI (MM-TBI), severe TBI and orthopedic injury patients with samples collected within 48 h from injury and compared to healthy volunteers. Eighteen and 20 miRNAs were observed in MMTBI and sTBI groups respectively and among these, 10 miRNAs were present at both TBI severities. Finally, four of these 10 miRNAs were also found in CSF (85). Di Pietro et al. screened 754 microRNAs using TaqMan Array Human MicroRNA A+B cards in mTBI+EC (extra-cranial injury), sTBI+EC, EC only groups and compared the results to healthy volunteers at different time points. Particularly interesting were the results obtained within the first hour from injury, in serum of mTBI+EC. These data reported two microRNAs, miR-425-5p and miR-502, having high diagnostic accuracy (AUC > 0.9) in differentiating mTBI from sTBI (84).

Recently, saliva was also explored as potential source of biomarkers for TBI. Salivary microRNA changes were found to be associated with prolonged concussion symptoms in pediatrics (86). Five miRs (miR-320c-1, miR-133a-5p, miR-769-5p, miR-1307-3p and let-7a-3p) were detected in the patients with prolonged post-concussive symptoms, and three of them; miR-320c-1, miR-629, and let-7b-5p were associated with memory problems, headache and fatigue that were developed 4 weeks after head injury. The same group has also matched miRNA changes in saliva and CSF, identifying six miRs (miR-182-5p, 221-3p, 26b-5p, 320c, 29c-3p, and 30e-5p) with similar changes in both biofluids (87).

A completed list of microRNA detected in different biofluids in TBI patients can be found in Table 2. Results presented, were not always consistent. However, it is not always possible to compare these studies, since sample collection timing or the different biofluid analyzed, play a relevant role to uniform the biomarker discovery.

**Table 2:** MicroRNA differentially expressed according the severity of TBI and the different human biofluid.

**Sample**	**microRNAs**	**TBI patients**	**References**
Plasma	miR-765, miR-16, miR-92a	Mild and severe	(81)
Plasma	miR-142-3p, miR-423-3p	Mild, moderate, and severe	(83)
Plasma	miR-23b	Severe	(88)
Serum	miR-1255b, miR-151-5p, miR-194, miR-195, miR-199a-3p, miR-20a, miR-27a, miR-27b, miR-30d, miR-328, miR-362-3p, miR-381, miR-486, miR-505*, miR-625*, miR-638, miR-92a, miR-451, miR-1291, miR-130b, miR-19a, miR-20a, miR-296, miR-29c, miR-339-3p, miR-579, miR-601, miR-660, miR-9*	Mild, moderate, and severe	(85)
Serum	miR-425-5p, miR-502, miR-21, miR-335	Mild and severe	(84)
Serum	miR-93, miR-191, miR-499	Severe	(82)
CSF	mir-9	Severe	(89)
CSF	miR-451, miR-328, miR-362-3p, miR-486	Severe	(85)
CSF	miR-141, miR-257, miR-181*, miR-27b*, miR-483-5p, miR-30b, miR-1289, miR-431*, miR-193b*, miR-499-3p, miR-1297, miR-33b, miR-933, miR-449b	Severe	(82)
CSF	miR-182-5p, miR-221-3p, miR-26b-5p, miR-320c, miR-29c-3p, miR-30e-5p	Severe pediatric TBI	(87)
Saliva	miR-182-5p, miR-221-3p, miR-26b-5p, miR-320c, miR-29c-3p, miR-30e-5p	Severe pediatric TBI	(87)
Saliva	miR-320c-1, miR-133a-5p, miR769-5p, miR1307-3p, let-7a-3p, miR629, let-7b-5p	Children with Post-concussion symptoms (PCS)	(86)

miRNA* = The RNA strand of the miRNA duplex that is complementary to the mature miRNA is shown with a star symbol (miRNA*).

Many microRNAs were also described in the brain of injured animals by using different models of TBI. Some of these studies have also investigated the potential pathobiology of the microRNAs differentially expressed in tissue.

Human miR-21 is one of the most studied miRs in TBI. It is a polycistronic miR (chromosome 17q23.2), and it overlaps with the Vacuole Membrane Protein 1 (VMP1) coding gene, also known as Transmembrane Protein 49 (TMEM49) (90).

Recent studies have demonstrated high miR-21 expression levels after TBI. Also, it has been found to improve the neurological outcome through inhibiting apoptosis and targeting angiogenesis molecules. In particular, the upregulation of miR-21 was found to reduce brain oedema derived by BBB-leakage. Hence, ago-miR-21 treatment was proposed as a potential therapy to decrease BBB damage (91) by inhibiting the loss of occludin and claudin-5 among other tight junction proteins. It also increases the levels of Angiopoietin-1 and its Tie-2 receptor, which maintain the normal BBB condition. MiR-21 was also found to improve experimental TBI mice cognition after the running wheel exercise (92, 103). The therapeutic role of miR-21 might also be due to inhibiting apoptotic cell loss by targeting the phosphatase and tensin homolog (PTEN)-Akt pathway (91). In an interesting study, extracellular vesicles (EV) were isolated from the brain of injured mice and controls, and the expression of miR-21 was found significantly increased with the injury. Concomitantly, an increase of miR-21 in neurons was observed, suggesting miR-21 secretion from neurons by EV cargo (92). Further support via the upregulation of miR-21 was also found in the serum of sTBI patients but not of mTBI, at very early time points and up to 15 days from injury. Also, no increase was found in the musculoskeletal injured patients, and for this reason, miR-21 was considered as a potential new TBI biomarker and a future therapeutic target for TBI (84).

Another exciting miR associated with TBI is miR-16, involved in the regulation of several biological processes activated after TBI; such as being involved in apoptosis by targeting BCL-2 (93) and in the cell cycle by targeting CDK6 (cyclin-dependent kinase 6), CDC27 (cell division cycle 27) and CARD10 (caspase recruitment domain 10) (94, 95, 148). Also, miR-16 was significantly increased within the first 24 h in the mild TBI patients and significantly decreased in the severe TBI patients compared to the healthy volunteers (81). MiR-107 was found to be underexpressed in cortex and hippocampus of a rat model of severe controlled cortical impact (CCI) (96). MiR-107 can regulate granulin (GRN) mRNA, suggesting a role in inflammatory process, energy metabolism and neuron regeneration (104). MiR-27a and miR-23a were downregulated in mouse cortex in a moderate model of CCI and was found to regulate pro-apoptotic Bcl-2 family members (97). Furthermore, miR-711, was upregulated in hippocampus after severe CCI, (96) and was found to reduce the neuronal cell death and lesion volume via Akt-pathway.Let-7i is another exciting biomarker with potential implications in TBI. It was upregulated in the serum and CSF of the rodent model of mild to moderate blast overpressure wave. It might be a potential regulator of many proteins and inflammatory cytokines, including S-100β and UCH-L1 (98). A detailed list of the pathobiology for miRNA differentially regulated in animal models of TBI can be found in Table 3.

**Table 3:** Pathobiology of the main differentially expressed microRNAs in brain of different animal injury models.

**microRNAs**	**Tissue**	**Animal injury model**	**Pathobiology**	**References**
miR-21	Cortex\ hippocampus	FPI/CCI	Apoptosis, dendritic spine morphogenesis, Angiogenesis, alleviating BBB leakage, cognition	(90–92, 99–103)
miR-107	Hippocampus	CCI	Neuron regeneration, inflammation	(96, 104)
miR-16			apoptosis, cell cycle	(93–95, 148)
miR-9	Cortex	FPI	Damaging the cytoskeleton and cellular integrity	(105)
mir-27b	Cortex	FPI	Disrupting amino acid and nucleic acid metabolic processes, hindering macromolecule complex assembly	(105)
miR290, miR-497	Cortex	FPI	Intracellular transport	(105)
mir-451	Cortex	FPI	Inflammation	(106)
miR-874	Cortex	FPI	Intracellular transport, apoptosis, inflammation	(105, 106)
miR-34a	Cortex\ hippocampus	FPI	Inflammation, apoptosis,	(106)
mir-144	Hippocampus	CCI	Synaptic function, cognition	(107)
miR-153	Hippocampus	CCI	Cognition	(107)
miR-23a, miR-27a	Cortex	CCI	Apoptosis	(97)
mir-155, miR-223	Hippocampus	CCI	Mitochondria associated miRs, inflammation	(108)
miR-711	Hippocampus\ cortex	CCI	Apoptosis	(96, 109)
let-7i	CSF\ serum	Blast	Regulator of inflammatory cytokines	(98)
miR-92a, miR-674, miR-138, miR-124, let-7c	Cortex	CCI	Behavior	(103)
miR-142-3p, miR-221	Hippocampus	CCI	Cell proliferation and angiogenesis of PDGF signaling pathways	(110)
miR-23b	Plasma\ hippocampus\ cortex	WDI	Reduce lesion volume of contused hemisphere and brain oedema, cognition	(88, 97)

## Biomarker discovery: MicroRNA profiling

Numerous studies investigated the global profiling of miRNAs in human diseases with the aim to identify a variety of biomarkers when compared the normal and affected tissues, which can further be correlated with the prognosis or the therapeutic response. MicroRNA can be extracted from a variety of sources, including cell lines, fresh tissues, formailin-fixed paraffin embedded (FFPE) tissues and also biofluids such as plasma, serum, urine, saliva and CSF.

**Figure 1:**
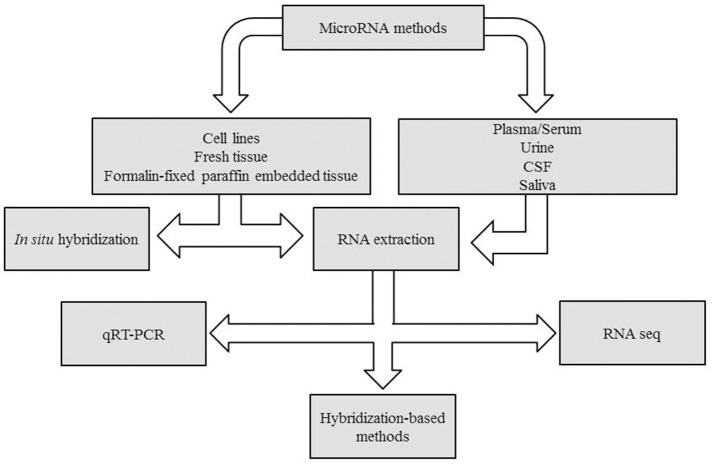
Decision making chart. MiRNAs can be extracted from different sample types, such as tissues and body fluids. The experimental design determines the methodology chosen for miRNA detection.

Many are the techniques used to analyze microRNAs. Generally, qPCR is suitable to investigate one or two miRNAs, whereas for larger studies examining multiple miRNAs at once, platforms such as TaqMan™Array Microfluidic Cards, miScript miRNA PCR Array or nCounter® microRNA panels, are more suitable. Finally, to discover new miRNA variants, the Next Generation Sequence (NGS) solution results are more appropriate. In Figure 1 a decision making chart is represented.

### Sampling

Sample processing and storage is the first step to perform miRNA profiling. This step is particularly crucial in order to obtain high-quality microRNA, especially for the determination of miRNA expression in biofluids. MiRNAs are stable in biofluids because of their molecular size and because they are protected within protein complexes or contained within EVs (microvescicles or exosomes). However, an immediate separation of cells is required to prevent lysis of cells and to avoid RNA contamination.

In addition, caution must be taken when collecting plasma or serum. Heparin-plasma for example, is a potent PCR inhibitor (111). Differentially, plasma-EDTA does not affect PCR and can overcome clotting due to platelet activation. In addition, plasma content of miRNA is higher than serum which is confirmed by slightly lower Ct value in the plasma (112).

### RNA extraction

Different kits are commercially available for miRNA extraction from different tissues or biofluids such as miRNeasy (Qiagen), mirVana TM (Ambion) or PureLink TM (Invitorgen) miRNA. The most commonly used kits are based on two main steps. The first one, is a chemical extraction with guanidine thiocynate (e.g., Trizol and QIAzol reagents); the second one, is an extraction procedure based on silica columns. New phenol-free kits were also recently developed such as ISOLATE II miRNA (Bioline) or ReliaPrep™miRNA (Promega).

Alternative strategies apply magnetic bead–based technology to purify samples, such as TaqMan™miRNA ABC Purification Kit (Thermo Fisher). All these kits differ, compared to those used for total RNA extraction, for additional steps to enrich the smallRNA fraction (113). Extraction from biofluid samples are particularly challenging, compared to extraction from tissues or cells, because of the lower RNA content, the possibility of hemolysis or platelet contamination and presence of serum proteins (such as RNases and PCR inhibitors). In addition the lack of well-established reference genes makes it difficult to analyze and interpret the data (114).

Nevertheless, several strategies can be used to maximize RNA yield. Among these, RNase-free glycogen, which acts as nucleic acid carrier can be added during the extraction (112). Similarly, other RNA carriers, such as the bacteriophage MS2 RNA, can also help to maximize RNA recovery (115). Therefore, monitoring the efficiency of the RNA extraction by addition of a known amount of synthetic miRNA spike-in is recommended (116). Alternatively, isolation of exosomes from biological fluid can help to increase the amount of retrieved RNA.

Exosomes are vesicles with diameter between 30 and 100 nm, originated from multivesiculated body (MVBs) and released into the extracellular space. The exosomes are able to carry different molecules as mRNAs, miRNAs, lipids and proteins and to transfer their contents to recipient cells, therefore influencing different physiologic and pathologic processes (117). The current techniques to separate exosomes from biological fluids include methods based on exosome size differences, as ultracentrifugation or size exclusion chromatography, or on identification of specific surface markers as immunoaffinity capture-based techniques (118). In ultracentrifugation procedure, the force used ranges from ~100,000 to 120,000 × g. After the centrifugation step, the exosome pellet is dissolved in phosphate buffered saline (PBS) and subjected to subsequent centrifugation runs with increasing force. Finally, isolated exosomes can be stored at −80°C until further analysis or in Trizol for RNA extraction. Based on their size, exosomes can also be purified by using membrane filters with 0.2 μm of diameter. This method, although widely used, can result in samples contaminated by others EVs and a large sample volume is requested. Other techniques were also developed to isolate exosomes. The presence of tetraspanins as exosomal surface markers, for example, is used for immunoaffinity reactions and different companies have already developed specific kit, based on affinity spin columns for exosome purification (Invitrogen, Qiagen).

### Quantification and quality control

The measurement of RNA concentration by using conventional spectrophotometers, such as nanodrop, is not possible for miRNA quantification and quality control (119). However, RNA integrity can be checked by spectrophotometry and automated capillary electrophoresis instruments such as the Bioanalyzer 2100 (Agilent) and Experion (Bio-Rad). In particular, the Bioanalyzer 2100, can also estimate miRNA concentration as the result of the ratio of 15-40nt RNA fragments and the total RNA, (120) providing that RNA integrity is very high. For this reason; it is a common practice to perform the analysis using established volume and not concentration of RNA extracted from the same volume of biofluid or tissue. However, accurate strategies to relatively quantify the sample are still necessary.

### miRNA profiling

The most widely and well established approaches used to determine microRNA profile can be divided into three main categories: quantitative real time PCR (qRT-PCR), hybridisation-based methods (i.e., Microarrays, Nanostring) and high-throughput next generation sequencing. The main advantages or disadvantages in using the above techniques are reported in Table 4.

**Table 4:** Advantages and disadvantages of the main microRNA profiling methods.

**Profiling methods**	**Time**	**Sample input**	**when to use it**	**Advantages**	**Disadvantages**
qPCR/Microfluidics	≤6 h	500/10 ng	small scale experiments large experiments also possible (multiwell dishes or microfluidic cards)	established protocol, high sensitivity and specificity, absolute and relative quantification, used for validation of large scale experiments.	intensive labor, requires quality miRNAs, relatively expensive, cannot identify novel miRNA.
Microarray	~2 days	100 ng−1 μg	large studies	established protocols, easy and fast, inexpensive.	less sensitive than qPCR, hard distinguishing similar sequences, no absolute quantification.
NextGen Sequencing	1–2 weeks	500 ng−5 μg	discovery phase	whole content analysis, single base resolution, not depending on any prior sequence knowledge, can detect low abundance transcripts, can detect new miRNAs.	equipment costs, bioinformatics support, less sensitive than qPCR, no absolute quantification.

#### qRT-PCR

qRT-PCR is the most popular technique to accurately assess miRNAs.

The single assay is primarily used to efficiently validate the results of large screening studies or for relatively small experiments.

The technique is relatively expensive and can be divided in two main steps: the conversion of miRNA into cDNA and quantitative polymerase chain reaction.

Because of the length of miRNAs and the lack of a common sequence such as a poly(A) tail that can be used for reverse transcription, cDNA synthesis presents its own challenges.

Two main strategies are used to generate cDNA:

1. The use of a stem–loop RT primer which first hybridizes with the miRNA strand, followed by reverse transcription using MultiScribe reverse transcriptase. cDNA products are then amplified using conventional TaqMan PCR.
2. The addition of a poly(a) tail using *E. coli* poly(A) polymerase (assay). An oligo-dt primer is then used to pair the miRNA tailed and allows the retro-transcription of the resulting cDNA, which is further amplified using specific primers and detected by the use of a fluorescent dye such as SYBER green.

However, large experiments using qRT-PCR can become quite laborious to perform. In order to overcome this problem, reactions can also be carried out in high-throughput form.

Pre-plated PCR primers, for example, are commercially available and distributed typically across multiwall dishes, or alternatively microfluidic cards containing nanoliter-scale wells. However, performing highly parallel qRT-PCR might present some challenge due to differences in primer annealing temperatures. However, it is still possible to solve this issue by using the locked nucleic acids (LNAs) into primers and allowing the optimal hybridisation conditions for several PCR assays to be run simultaneously (114).

qRT-PCR allows both absolute and relative quantification. In the first one, a standard curve from serial dilutions of known concentrations of synthetic miRNA is generated and used to calculate the number of copies of a specific miRNA. In the second case, before setting up the microRNA expression analysis, an endogenous normalizer (reference gene) has to be chosen, among several control candidates tested. These candidates offer stable expression over the whole range of samples, and are selected based on the literature or pre-existing data.

Hsa-miR-16-5p is widely used in the literature as an endogenous miR, despite the lack of a panel of endogenous miRNA consensus (121). Hsa-miR-223 (116) hsa-let-7d-5p (122) hsa-miR-484 (123), hsa-miR-191-5p (124), and hsa-miR-423 (125) were also described as relatively invariant reference genes in plasma/serum. MiR-331 and miR-223 were identified as the most stables in traumatic brain injury patients (84). MiR-202 was also used as normalizer gene in CSF of TBI patients (85).

In addition, to identifying the appropriate endogenous controls, it is also possible to use some software as geNorm Algorithm (https://genorm.cmgg.be/) and DataAssist v.3 software (Applied Biosystems). GeNorm is used to normalize the data from a large and unbiased set of miRNAs. DataAssist is useful to quantify gene expression in samples when using the comparative CT (ΔΔCT) method (126, 127). However, it is always preferable to add a spike-in control during the RNA extraction and to normalize the microRNA using an exogenous control (e.g., cell-miR-39).

#### Hybridization-based methods

Several hybridization-based methods exist to identify microRNA abundance. *In situ* hybridization (ISH) is the most used method to localize DNA or RNA using labeled complementary nucleic acid probes in tissue section or fixed cells (128). However, this technique is not suitable for miRNA detection because of their length, but the introduction of LNA showed a significant improvement in the sensitivity and specificity of this technique applied to miRNAs detection (129). Microarray-based technique is another powerful high-throughput method extensively used for microRNA profiling, because of their ability to screen large number of miRs simultaneously in large variety of samples (from tissue to biofluid). MiRNA microarray is a nucleic acid hybridization technique which uses amino-modified 5' termininal complementary probes immobilized onto glass slides through covalent crosslinking between the amino-groups and the self-assembling monolayer (130). After RNA purification, miRNAs are tagged with fluorophore-labeled nucleotides at their 3′ end. LNAs can also be incorporated into capture probes to increase specificity and sensitivity (131). The main advantages of using micrarray are the low costs and the parallel measurements. Typically microarray involves a comparison between two or more groups and cannot be used to determine absolute quantification. Because of limited specificity, data obtained are typically validated by a qRT-PCR.

A new technology, the Nanostring nCounter Analysis System, was recently developed to allow the quantification of more than 800 RNA molecules in 12 samples, in a single assay. The nCounter Analysis System is a very new technology which uses digital color-coded barcode for precise multiplexed measurement of the gene expression (<1 copy per cell). This system is more sensitive than microarrays and as sensitive and accurate as qRT-PCR. The combination of color-coded barcode attached to a single target-specific probe corresponding to a gene of interest and the single molecule imaging, allows detecting and counting hundreds of unique transcripts in a single reaction. Each color-coded barcode represents a single target molecule. No amplification is required (132).

Finally, to identify the significant differentially expressed miRNAs in a large genomic data, such as the microarray data but also the microfluidic card and the RNA sequencing data, the most frequently used method is the Significance Analysis of Microarrays (SAM) computed by Multi Experiment Viewer (MEV) v4.8.1 (http://www.tm4.org).

#### RNA sequencing

The introduction of the next generation sequencing has become increasingly popular in biomedical research, overcoming the limitations of the microarray analysis (133). While it cannot quantify miRNA levels with the same resolution of qPCR, it still has the advantage to detect all known or unknown miRNAs present in a sample and to precisely distinguish all isoforms in the absence of background and cross-hybridization problems. IsomiRNAs, indeed are miRNA containing sequence variations, typically by shortening or lengthening of the 3′ end. Over 3,300 miRNA variants were identified and reported at the following website http://galas.systemsbiology.net/cgi-bin/isomir/find.pl. However, one or two isomers contribute to >90% of the signal detected, while the remaining variants are not abundant enough to be revealed.

The procedure consists in a small-RNA cDNA library preparation followed by “massive parallel” sequencing on a single run. First of all, miRNA fragments are extracted from total RNA. Running the sample on an agarose gel and cutting out the band corresponding to the miRNA size is the second step. Then, the selected RNA fragments are ligated to sequencing adapters and transcribed into cDNA by ~12–15 RT-PCR cycles of amplification and using a reverse transcription primer which hybridizes to the 3′ adapter.

At this point, another run on agarose gel of the obtained cDNA library is performed and the band with size corresponding to the length of adapter sequences plus the miRNA insert of ~20–30 bases (for a total length of 120 bp) is cut out and ready for sequencing. The gel size selection is particularly crucial because of the potential presence of adapter dimer side products created during the ligation step as well as higher molecular weight products generated from ligation of other RNA fragments, such as tRNA and snoRNA, containing 5′ phosphate groups.

Significant computational resources and bioinformatics expertise are required for data interpretation not only for known miRs but also for the newly discovered miRs. Initially all generated reads are aligned to the reference genome of the sequenced organisms. Short read aligner tools are available to process the reads such as maq (http://maq.sourceforge.net/maq-man.shtml), sop (http://soap.genomics.org.com) or bwa (http://bio-bwa.souceforge.net/). In addition, it is also important to filter out reads that align against other non-coding small RNA species and RNA degradation products which sequences are available on the University of Santa Cruz (UCSC) Genome Browser.

Another bioinformatics challenge is the relative quantification. Expression levels are analyzed on the base of the read counts for each sequenced sample. The number of reads of each individual molecule is normalized against the total number of reads produced in the same sample (134).

Different tools are also available to predict novel miRNAs from generated data. One of the commonly used is mirDeep (https://www.mdc-berlin.de/8551903/en/) (135).

Although the NGS is one of the most advanced techniques currently used, other challenges, beside the bioinformatic support, need to be faced. One of these is the cost required for equipment, software and consumables. In addition a high quality of purified RNA and a large amount of RNA, usually 5 μg, are required for the analysis. Validation is another important aspect to address in order to use this technology for diagnosis and prognosis of diseases.

### MicroRNA database and target prediction

Since miRNAs control the regulation of several genes and they are linked to many disorders, it is also possible to reliably predict potential miRNA targets which can be involved in these pathologies. The prediction of the mRNA targets is based on the partial complementary sequences between the mature miR and the mRNA candidate target. This search is generated by miRNA target prediction algorithms able to seek for putative binding sites in the 3′UTRs of the candidate mRNAs (i.e., PicTar, TargetScan, DIANA-microT, miRanda, rna22). High complementarity between the miRNA and the target binding region results in the degradation of the target, whereas the presence of mismatches represses the translational process. However, results of their applications are often not consistent and must be experimentally validated. Many lab-based techniques can be used to overcome the challenge of target validation such as the inverse correlation between the expression of miRNA and its target, the effect on protein expression /function or a direct validation by using the luciferase assay or their functional effects (proliferation, differentiation, apoptosis) on a cell culture system.

In addition, databases such as mirTarBase or miRewcords collect both predicted and experimentally confirmed miRNA targets.

Finally, functional analysis of miRNAs or miRNA high-throughput data sets can also been performed. For example, Gene Ontology (GO) analysis is commonly used to identify pathways and processes from a list of genes provided, for example from results obtained using gene expression microarray (136) or generated from a target prediction tools in the case of miRNA (137).

## Limitations in the use of miRNAs as circulating biomarkers

The use of miRNA signature as a novel diagnostic/prognostic tool is still in the descriptive phase. Numerous data have been collected so far, in various disease states; however their translation in clinical applicability requires much larger studies and universally implemented guidelines.

First of all, the lack of methodological details in published papers makes it difficult to directly compare the results, and lead to inconsistent or even contradictory results.

Standard protocols must be achieved for the different steps of miRNA analysis such as sample processing, RNA extraction and expression measurement/assessment methods as well as differences in specimen type, for example FFPE *vs*. fresh frozen samples, must be considered.

In addition, the research of miRNA profile in biofluids is particularly challenging as miRNAs, circulate either associated with proteins, lipoproteins or EVs, and this might require specific precautions during the extraction or analysis processes. Moreover, it is good practice to check the presence of small clots and hemolysis in plasma/serum which may contribute to the variability in miRNA expression.

Furthermore, we are not aware if miRNA expression varies at specific conditions such as: fasting or circadian rhythm, thus, standardization and annotation of these protocol details is necessary in order to minimize variability of unknown factors.

Data normalization, identification of well-characterized endogenous miRs specific to biofluid and pathology of interest, as well as characterization of baseline levels for miRs described as potential biomarkers are other crucial points in obtaining accurate results.

Certainly, a common information infrastructure for data exchange, analysis and protocols used would facilitate research in the miRNA biomarker discovery.

## Point-of-care diagnostic tools to detect circulating microRNAs as biomarkers of disease

Besides the challenge of biomarker discovery, there lies the challenge of rapidly detecting them with clinically relevant sensitivity and specificity using a low-cost and easy point-of-care injury test.

In the case of traumatic brain injury, a PoC technology would have several applications. This is particularly true for mTBI which represents a serious problem in military, and contact sports that has led to reduction in the sport participation in younger age groups.

The development of a pitch-side or “pre-hospital,” portable TBI diagnostic devices, would implement the current guidelines in the management of mTBI (17, 138) in different ways:

1. In the initial pre-hospital assessment to determine whether patients should be transferred to a Major Trauma Centre or a local Trauma Unit.
2. In the Emergency Department (ED), to determine the need for a CT brain scan.
3. Pitch-side, to assist decision making as to removal from play and assessment of the need to take the player to the ED.
4. In sports clinics, to diagnose a concussive event and guide return to play.
5. In combat theaters, to determine the need to dispatch a rescue team.

So far, proteins were widely explored as biomarkers and immunoassays are extensively used as method of detection, although not often very sensitive and prone to false positives (139).

The PCR amplification method has played an important role in diagnostics over the last years because of its ability to detect few molecules (140) and the fact that microRNAs are particularly stable in biofluids, positions them as a new valid potential biomarkers to explore. MicroRNAs are also particularly suitable for these clinical applications as they are molecular switch regulators and for this reason their early expression anticipates the molecular mechanisms trigged by TBI.

Several companies are now working on point-of-care device that can measure microRNAs in the field. This is quite challenging, although not insurmountable, as microRNAs are present in femtomolar and picomolar concentrations and need to be extracted from the biofluid first.

Micorfluidics is another challenging problem. Transporting the methodology in a portable device, reducing the volume to few microliters over a few millimeters and mixing the rinsing solutions and all reagents are main issues.

Detection is another important point to discuss; various strategies were developed to improve the detection of miRNA (141).

Nanoparticles(NPs)-based biosensors, for example, are widely studied. The use of this biosensor has the potential to miniaturize the equipment and reduce the cost. In particular, carbon and metal-based NPs, such as gold nanoparticles (AuNPs) are excellent miRNA carriers that can be used to accelerate the signal transduction enhancing a rapid analysis and lowering the detection limit. Recently, a dual-function gold nanoparticle biolaben was used to detect miR-21 in serum (142).

Magnetic nanoparticles are also very popular. Wanunu et al. (143) developed a protocol using probe:miRNA duplex binded to p19-functionalized magnetic beads, which are first eluted and electronically detected using a nanopore (143).

Optical detection in combination with NP probes was also explored in the development of a novel highly specific and reproducible platform, the Scanometric MicroRNA (Scano-miR), to detect low concentrations of miRNAs (144).

Surface plasmon resonance (SPR) biosensors, is another example of label-free optical biosensing technologies. This method is based on optical measurement of refractive index changes given by the binding of analyte molecules present in samples to specific receptors immobilized on the SPR sensor. This method showed to be able to detect miRNA in <30 min at concertation down to 2 pM (145).

Finally, enzyme catalytic amplification-based electrochemical assay are also developed for this purpose (146).

However, hard work is still required to develop a reliable portable PoC device.

## Conclusions and perspectives

miRNA profiling and detection provide valuable information on their essential roles in normal cellular function and disease, projecting their use in the clinical practice for the diagnosis and prognosis of several pathologies. With this review, our aim was to provide insights into the miRNA expression in TBI, the main commonly used detection methods to discover new biomarkers and the state-of-the art of the PoC development.

Despite their limited use as routine biomarkers, several companies already offer miRNA-based diagnostic assays.

In addition, there are new emerging classes of non-coding RNA such as piwi-interacting RNAs, and long non-coding RNA (lncRNA) that have important role in cellular physiology.

In the future, profiling methods that have the potential to detect all the RNA classes are likely to improve the understanding of the whole transcriptome and provide new valid information for the diagnosis, prognosis and therapy of several pathologies, including TBI.

## Author contributions

VD drafting the article and final approval of the version to be published. KY drafting the article. US drafting the article. CD critical revision of the article. AB critical revision of the article.

### Conflict of interest statement

The University of Birmingham has intellectual property associated with miRNA listed in this manuscript. The authors declare that the research was conducted in the absence of any commercial or financial relationships that could be construed as a potential conflict of interest.
